# Strongly enhanced light trapping in a two-dimensional silicon nanowire random fractal array

**DOI:** 10.1038/lsa.2016.62

**Published:** 2016-04-08

**Authors:** Barbara Fazio, Pietro Artoni, Maria Antonia Iatì, Cristiano D'Andrea, Maria Josè Lo Faro, Salvatore Del Sorbo, Stefano Pirotta, Pietro Giuseppe Gucciardi, Paolo Musumeci, Cirino Salvatore Vasi, Rosalba Saija, Matteo Galli, Francesco Priolo, Alessia Irrera

**Affiliations:** 1 CNR-IPCF, Istituto per i Processi Chimico Fisici, 98158 Messina, Italy; 2 Dipartimento di Fisica e Astronomia, Università di Catania, 95123 Catania, Italy; 3 CNR-MATIS IMM, Istituto per la Microelettronica e Microsistemi, 95123 Catania, Italy; 4 Dipartimento di Fisica, Università degli Studi di Pavia, 27100 Pavia, Italy; 5 Dipartimento di Scienze Matematiche e Informatiche, Scienze Fisiche e Scienze della Terra, Università di Messina, 98166 Messina, Italy; 6 Scuola Superiore di Catania, Università di Catania, via Valdisavoia, 9, 95123 Catania, Italy.

**Keywords:** light trapping, multiple scattering, Raman enhancement, random fractal, silicon nanowires

## Abstract

We report on the unconventional optical properties exhibited by a two-dimensional array of thin Si nanowires arranged in a random fractal geometry and fabricated using an inexpensive, fast and maskless process compatible with Si technology. The structure allows for a high light-trapping efficiency across the entire visible range, attaining total reflectance values as low as 0.1% when the wavelength in the medium matches the length scale of maximum heterogeneity in the system. We show that the random fractal structure of our nanowire array is responsible for a strong in-plane multiple scattering, which is related to the material refractive index fluctuations and leads to a greatly enhanced Raman scattering and a bright photoluminescence. These strong emissions are correlated on all length scales according to the refractive index fluctuations. The relevance and the perspectives of the reported results are discussed as promising for Si-based photovoltaic and photonic applications.

## Introduction

The development of new materials for light trapping, emission and amplification of light is an ever-growing research field. Novel concepts of thin films textured at the micro- and nanoscale, and assemblies of nanostructures with peculiar spatial arrangements, both ordered and disordered, have a key role on the light transport inside the materials and, consequently, on their optical properties^1, 2, 3, 4, 5^. Recently, a new strategy of designing two-dimensional (2D) random patterns of submicron size holes in thin films has been demonstrated^6, 7^. These new structures allow for strong and broad optical resonances, leading to in-plane multiple scattering phenomena, efficient light trapping and absorption enhancement beyond the theoretical limit dictated by ray optics^8, 9, 10^. In this scenario, the production of a fractal pattern presents the possibility of achieving a complex disorder with strong structural heterogeneities correlated on all length scales^11, 12, 13^. Recently, plasmonic fractal-like structures have been proposed to improve photovoltaic device performances; indeed, through an efficient coupling of the incident light at different frequency bands into both the cavity modes and the surface plasmon modes^14^, a broadband absorption enhancement can be reached^15^.

Alternatively, high refractive index-textured materials, in particular semiconductor nanostructures^16^ and nanowires (NWs)^17, 18, 19^, are good candidates to scatter, trap and localize light, minimizing the parasitic optical losses typical of metallic structures^20^.

Currently, silicon is certainly the most important and well-known semiconductor because Si-based devices have dominated microelectronics for many decades. Indeed, the production of smart Si NW materials to enhance light scattering and absorption is currently the most convenient approach. In particular, ‘black-silicon’, which is obtained through the exposure of a crystalline silicon surface to a reactive-ion etching (RIE) process with various gases^5, 21^, has been reported to exhibit exceptional antireflection properties, with extremely low reflectance values (below 1%) and high absorbance over a wide spectral range in the visible and near-infrared regions. This type of material exhibits conical-shaped needles with typical cross sections varying from a few to hundreds of nanometers. These needles act as an almost perfect graded index layer on the Si surface, thus strongly suppressing reflectivity of the incoming light regardless of the propagation direction^5^. Moreover, strong light-trapping and absorption properties in terms of an enhanced short-circuit current have also been demonstrated in Si NW solar cells, leading to a path-length enhancement exceeding the randomized scattering Lambertian limit^22, 23, 24, 25^. Our approach involves the fabrication of a 2D random fractal structure of Si NWs as a unique material that meets all of the previously described demands. Here, a forest of ultrathin and vertically aligned silicon NWs, arranged in a 2D fractal array, is reported; this array is obtained via a silicon-compatible technology without the use of any mask or lithographic process. Despite their ultra-small dimensions (only a few microns long with a diameter of few nanometers), this type of fractal NW array allows for a very high light-trapping efficiency across the entire visible and near-infrared range, reaching high values of apparent absorbance due to light absorption driven by multiple scattering inside the structure. Furthermore, these internal multiple scattering processes are responsible for a strong photoluminescence (PL) and an enhanced Raman scattering from the Si NW forest, paving the way toward a new class of light-emitting devices. We demonstrate that the strong Raman enhancement is strictly correlated to the length scales at which the refractive index primarily fluctuates.

## Materials and Methods

### Sample fabrication

Si NWs were prepared by using *n*-type Si substrates (111) and (100) for samples 1 and 2, respectively, with a resistivity of 1.5 Ω cm and thickness of 540 μm (350 μm in the case of polished back surface). Each substrate was oxidized and then dipped in hydrofluoric acid 5% to produce an oxide-free Si surface. A 2-nm gold layer was deposited onto the Si substrates at room temperature via electron beam evaporation (electron beam evaporator from Kenosistec s.r.l., Binasco MI, Italy) using high purity (99.9%) gold pellets. Si NWs were formed by etching samples in an aqueous solution of hydrofluoric acid (5 M) and H_2_O_2_ (0.44 M). The Au was removed via a KI dip^26^.

Plan view and cross-section images of the Si NW array were obtained using a field-emission scanning electron microscope (SEM) Zeiss Supra 25 (Oberkochen, Germany).

### Optical characterization

The diffuse (hemispherical) reflectance spectra were measured in the spectral range of 200–1800 nm by means of a double-beam spectrophotometer (Varian, CARY 6000i) equipped with an integrating sphere. In the used reflectance geometry, both the specular and diffuse components are sent to the detector in the integrating sphere.

Micro-Raman spectra were acquired using an HR800 Horiba—Jobin Yvon spectrometer in the back-scattering configuration. This setup allows for a multi-wavelength excitation, making use of a He–Ne laser, a diode laser emitting at 785 nm and two argon-ion lasers, one used to produce the Ultra-violet line at 364 nm and the other one for the visible wavelengths. For the detection of the Raman spectra, the excitation powers were maintained to be very low (ranging between one hundred and tens of microwatts, depending on the laser wavelength) by using a 100 × objective (numerical aperture (NA)=0.9 in air). For the PL measurements, we carefully focused the same excitation power for all different wavelengths used (by a 100 × objective with NA=0.9 in air). We acquired five sets of measurements (Raman and PL signals for each excitation wavelength) in different points of the sample to determine the trends. For each set of measurements, we examined exactly the same sample portion to properly evaluate the trend as a function of incident wavelength on the same structure. The error bars of Raman enhancement per volume (REV) and PL intensities values reflect the imprecision in setting the measurements and, more importantly, the statistical fluctuations of different points of the sample around the ensemble average. Because the Si NW material presents many holes, aiming to draw out the PL excitation (PLE) trend, we scaled the obtained intensity values for the objective focal depth and spot area calculated as a function of incident wavelength. By contrast, the Raman intensities were scaled for the different objective focal depths of both the laser excitations and the collected Raman wavelengths. It was not necessary to scale the values for the different objective spot areas because we extracted only a Raman enhancement normalized to the bare crystalline silicon measurements performed using the same experimental set up.

## Results and Discussion

**Figure 1:**
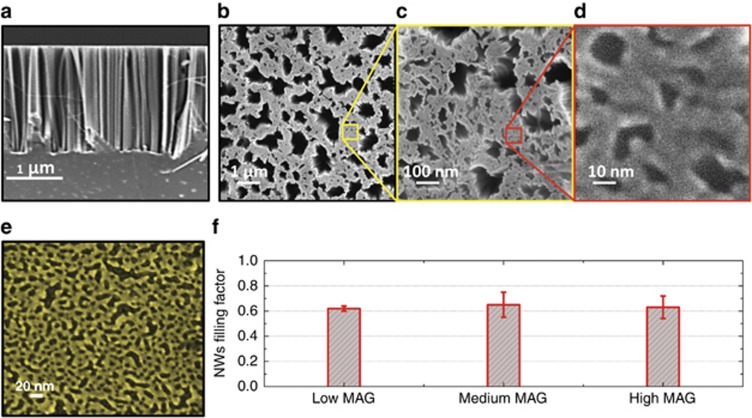
SEM images of Si NWs obtained by the metal-assisted wet etching technique. (**a**) Cross-section SEM image of Si NWs obtained by the metal-assisted wet etching technique. (**b**, **c** and **d**) Plan view SEM images of a Si NW sample obtained at three different magnifications (25 k ×, 250 k × and 2500 k ×). The structure is arranged in a Russian-nesting-doll-like distribution: in particular, panel c is the higher magnification of the sample area inside the yellow square in panel b, and panel d is the higher magnification of the sample area inside the red square in panel c. (**e**) Plan view SEM image of interconnected gold film deposited on a Si (111) surface. (**f**) Silicon surface coverage histogram for the plan view SEM images shown here.

The Si NW sample realized in this work on a Si (111) substrate is presented in Figure 1. Here, a forest of vertically aligned NWs, all having the same length (2.6 μm), appears (Figure 1a). The impressive self-similarity properties of the planar morphology is revealed in Figures 1b–1d. In particular, by analyzing the scanning electron microscopy plan view images, the surface coverage is estimated to be ~60% for all of the three different magnifications (Figure 1f), indicating scale invariance in the high-density 2D arrangement of the Si NW forest (evaluated to be 1.5 × 10^12^ cm^−2^). This particular texture is obtained after the deposition process of a thin gold layer (Figure 1e), the structure of which is close to the 2D percolation threshold (gold filling fraction 54.6%) on a silicon (111) surface. It is well known that an infinite cluster is a fractal object in the vicinity of the percolation threshold^27, 28, 29^. The gold deposition morphology is imposed on the silicon substrate as a negative mask during the wet etching procedure^26^; as a consequence, the Si NW distribution is organized with this specific structure.

**Figure 2:**
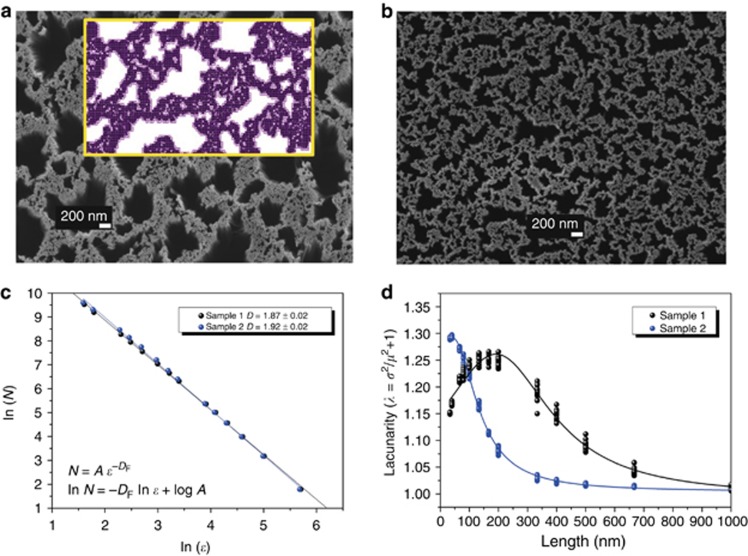
Characterization of 2D random fractal structures of Si NWs. Si NW samples 1 (**a**) and 2 (**b**) plan view SEM images used for Fraclac analysis. Details of the analysis with sectioning in square boxes in the reduced sample portion are shown in the inset of panel a. The fractal dimension and lacunarity plots obtained as analysis results are shown in (**c** and **d**), respectively. Note that the pixel size is 6.7 nm. Hence, length (nm)=*ε* (pixel) × 6.67 (nm per pixel).

To verify the fractality of the obtained Si NW sample, we calculated the fractal dimension of the structure. The planar arrangement of the NWs was studied using a top-down approach, which consisted of sectioning a highly resolved low-magnification image in square boxes of decreasing dimension; the analysis was performed on an extended sample portion (Figure 2a). Details of a reduced sample portion are shown in the inset of Figure 2a. The number of boxes *N* mapping the structure (pixels occupied by filled spaces) was measured as a function of the box size *ε* by using ImageJ software and the Fraclac plugin^30^, as shown in Figure 2c. Next, we obtained the fractal dimension *D*. The method produced a value for *D* of 1.87, corroborating the claim of a dense planar arrangement of the Si NW forest, which is exactly what we expected starting from a 2D percolation for an invasion cluster of gold^31^. This result was confirmed by an alternative bottom-up approach consisting of mapping the 2D array images in Figures 1b–1d through the iterative repetition of a planar model building cell until we covered the entire area of interest. The methodology used and the fractal dimension calculation procedures are shown in Supplementary Information Section S1.

In general, strong scattering manifests when the dimensions of the inhomogeneity are on the same scale as the effective wavelength *λ*_eff_ propagating in the medium, where *λ*_eff_=*λ*/*n*_eff_ with *n*_eff_ the effective refractive index. Infinite fractals obey the scale symmetries and are correlated on all length scales; thus, they are characterized by the absence of a characteristic length for the inhomogeneities. As a consequence, it is always possible to match the dimensions of the inhomogeneity regardless of the selected excitation wavelength^13^. However, our sample shows the structural characteristics of a finite fractal, having a cutoff value in the maxima dimensions of holes in the range of 1 μm. Thus, we expect that the wavelengths in the visible range have the highest probability to be matched. The fractal parameter that takes into account both the gaps and the heterogeneity of the structure is the lacunarity, defined as the measure of how the space is filled, being related to its gappiness (alternation of full and empty space)^32^. Lacunarity was calculated from the pixel distribution of the image shown in Figure 2a, and it was based on the variation in pixel density at different box sizes during the standard fixed non-overlapping box counting scan (details in Supplementary Information Section S2).

As observed in Figure 2d, our silicon NW material on Si (111) (hereafter sample 1) shows heterogeneities at all of the investigated length scales, as expected for a finite fractal system such as our NW array; indeed, the sample presents a lacunarity over a wide-length range. Moreover, a maximum of lacunarity is revealed on the scale of 150–200 nm along the structure planar section; this spatial range is then correlated with the strongest fluctuations of refractive index. Thus, we expect that the multiple scattering processes across the fractal structure at this length scale will be maximized. For the sake of comparison, we analyzed a different Si NW fractal sample obtained using a (100) Si substrate and the same etching process reported here (Figure 2b). The change in the silicon substrate orientation determines a different clustering dynamic^33^ that produces a variation in the morphology of the Au thin layer. In turn, the formation of Si NW fractal array shows *D*=1.92 in the plane and lacunarity that is always increasing at very small length scales (sample 2 in Figure 2c and 2d, respectively); note that the lacunarity analysis is halted at 33 nm.

**Figure 3:**
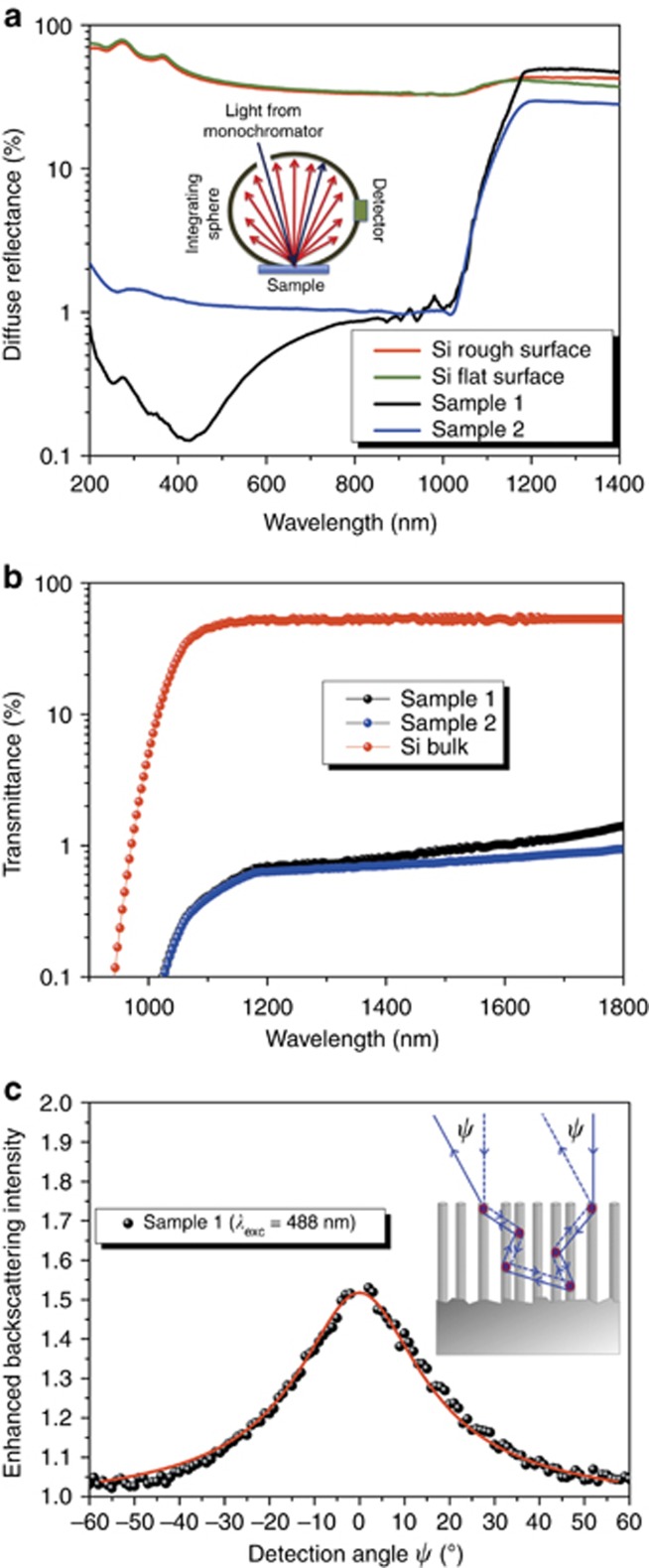
Light-scattering properties of Si NW random fractal array. (**a**) Diffuse (hemispherical) reflectance of Si NW sample 1 (black lines) and sample 2 (blue line) and of a bulk *c*-Si flat (front) and diffusive rough (back) surface (green and red lines, respectively). (**b**) Direct transmittance, by excluding the diffuse component, of samples 1 and 2 obtained in a polished back surface (black and blue dots, respectively) and of a bulk *c*-Si (red dots). (**c**) Coherent backscattering cone (black dots) of sample 1 obtained at an excitation wavelength of 488 nm; the red continuous line is the best-fitting curve obtained by the semi-infinite slab model. The inset shows a schematic sketch of the mechanism.

To study the light-scattering properties of our Si NW fractal forest, we first measured the diffuse (hemispherical) optical reflectance from the ultra-violet-visible to the near-infrared spectral range. Figure 3a shows the diffuse reflectance in four different cases: (i) Si NW sample 1; (ii) Si NW sample 2; (iii) optically flat Si sample; and (iv) optically rough Si sample, which we chose as the back surface of a silicon wafer, having an r.m.s. roughness in the range of a few microns. In the optically rough Si sample, the diffuse reflectance assumes the values expected from the average angle-dependent Fresnel coefficients at the Si–air interface, indicating a diffusing behavior mainly due to single scattering (such as reflectance) over all of the investigated spectral range. A radically different behavior is observed in the diffuse reflectance from the Si NW samples, which display a sharp drop to near zero (in the range of 1% for both Si NW samples) across the entire visible-NIR range for wavelengths just below the Si bandgap at 1.1 μm. This observation points to a strong absorption of the reflected light due to multiple scattering within the NW layer. We observed that a broadband antireflection behavior, similar to what is observed in many black silicon materials, can be excluded here because of the lack of a graded index profile along the height of the NWs. Indeed, as shown in Figure 1a, our Si NWs are characterized by a constant cross-section and an almost perfect vertical alignment, which lead to a constant average index of the NW layer along the vertical direction. Moreover, the large diffuse reflectance observed in the transparency region, that is, for wavelengths just above the Si bandgap, followed by the sharp decrease in the absorbing visible range, is incompatible with an antireflection effect due to a graded index profile, which would give a very smooth and low reflectivity across the Si bandgap as reported for black Si^5^. To further exclude an antireflection effect, as shown in Figure 3b, we measured the direct transmittance (by excluding the diffuse component) of our Si NW samples compared with that of a bulk Si wafer (both samples have a polished back surface). A very strong suppression of the transmittance to values below 1% is observed for both Si NW samples, which once again is incompatible with an antireflection effect by the NW layer because this would increase the transmittance compared with bulk Si instead of suppressing it. Note that we choose to show the transmittance measurements only in the near-infrared, because the values drop to zero across the entire visible range. According to these observations, we conclude that light is strongly diffused within the thin NW layer. In particular, in the strongly absorbing visible region, light is neither reflected nor scattered out of the Si NW forest but remains mostly trapped in the NW layer by multiple scattering processes until it is eventually absorbed (we suppose that the scattering processes are preferentially in-plane because the fractal shape and the refractive index fluctuations are across the 2D planar structure). A key role in this behavior is played by the particular 2D random fractal texture of our NW forest. Indeed, even if the ultra-small average diameter of our Si NWs (a few nanometers)^26^ could hardly lead to efficient light scattering by a single NW, the peculiar random fractal arrangement made of tightly spaced NWs separated by air voids (as shown in Figure 1) introduces strong heterogeneities and consequently highly efficient in-plane scattering pathways on a length scale between 10 and 1000 nm. Thus, one can think of light as scattered by ‘regions’ of NWs with varying densities rather than by single NWs. When multiple scattering occurs, the light path-length increases substantially, thus increasing the likelihood of absorption at the end^34^. Furthermore, observing in detail the reflectance spectra of the Si NWs (Figure 3a), we observed in sample 1 a large and broad minimum peak at ~428 nm and approaching 0.1%, which indicates light over-trapping by the structure. These very low reflectance values, already reported in the literature for some black silicon-based materials^5^, place our structure among the most appropriate silicon-based architectures for applications in solar cells, being also very inexpensive and easily implementable over a large area. The wavelength of the over-trapping feature corresponds to an effective wavelength in the medium *λ*_eff_ of 192 nm (*λ*_eff_=*λ*/*n*_eff_, with the refractive index *n*_eff_=2.23, as calculated by the Bruggeman mixing rule, assuming the following composition: 40% air voids, 40% silicon and 20% silicon oxide due to the native oxide on the Si NW surface)^35, 36^. This wavelength perfectly matches the length range at which the lacunarity shows its maximum value (Figure 2d), corroborating the hypothesis, whereby the higher the in-plane multiple scattering is, the higher the trapping efficiency is in the Si NW material. By contrast, no ‘over-trapping feature’ resonant with the structure is visible in sample 2, which shows the lacunarity shape increasing toward very small length scales out of any possible wavelength matching in the ultra-violet-visible range. This correspondence shows how a morphological property such as lacunarity is the main factor in the optical response related to light-scattering phenomena in these materials. We also investigated computationally^37, 38, 39, 40^ the relationship between the heterogeneities and the minimum position in the total reflectance curve in sample 1, building a simple random structure to simulate the regions of tightly spaced NWs separated by air voids. The results, shown in Supplementary Information Section S3, highlight the key role of the edge-to-edge distances between the nearest air voids, along with the alternation of full and empty space, in reproducing the reflectance experimental curve. Indeed, the over-trapping feature is better approached when these edge-to-edge hole distances match the effective wavelength in the medium. Furthermore, analyses on the specular reflectance (shown in Supplementary Information Section S4) highlight how the light-trapping property of the Si NW fractal sample is not affected by the angle of the incident radiation with respect to the sample surface.

To assess the scattering strength of our Si NW sample 1 in a more quantitative manner, we performed experiments of coherent backscattering of light (see Figure 3c). Coherent backscattering is a phenomenon of light transport in disordered media in which partial waves traversing time-reversed (momentum-reversed) scattering paths interfere constructively in the exact backscattering direction^41, 42, 43^, as illustrated schematically in the inset of Figure 3c. Departing from the backscattering direction, a rapid dephasing develops between the counter-propagating waves due to the configuration averaging of the path lengths in the medium. This gives rise to a typical cone shape in the angular-dependent scattered intensity. From the analysis of this feature, we obtained a transport mean free path *l*_t_ of ~160 nm when the exciting laser line is 488 nm; this value *l*_t_ places our Si NWs among the strongest scattering materials to date^44^.

**Figure 4:**
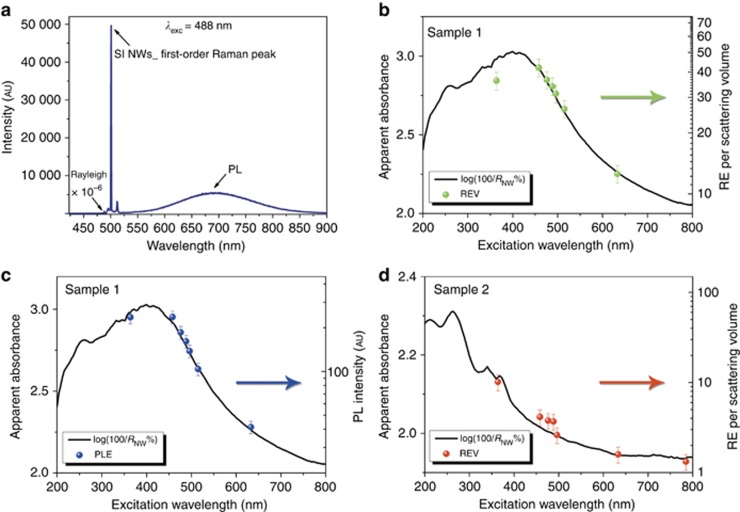
Photoluminescence emission and Raman scattering from the Si NW fractal array. (**a**) Raman backscattered radiation and PL emission from the Si NW sample 1 at an incident laser wavelength of 488 nm (power 20 mW on a spot with a 100 μm diameter). (**b**) Raman enhancement of Si NW sample 1 with respect to the *c*-Si bare substrate. (**c**) PLE of sample 1 evaluated at 690 nm. (**d**) Raman enhancement of Si NW sample 2 with respect to the *c*-Si bare substrate. All the trends are plotted as a function of the incident laser wavelength and compared to the apparent absorbance of the corresponding sample (black lines). The arrows indicate the *y*-axis corresponding to the plotted data having the same color.

Fractals are known as systems promoting electromagnetic field localizations because the property of dilation symmetry and the lack of translational invariance lead the structure to spatially localize the running waves, which are not eigenfunctions of the dilation symmetry operator^13^. Moreover, as already mentioned, a random fractal pattern is characterized by a self-similarity for which the structural heterogeneities are correlated on all length scales. As a consequence, whatever the effective wavelength propagating inside the medium, there will always be the possibility to match the length scale where the refractive index fluctuates and thus the possibility to generate a strong scattering. In some cases, this behavior can lead to inhomogeneous localizations of the electromagnetic field where both spatially localized and delocalized modes coexist^14, 45^. This typical property of a fractal structure results in the formation of hot-spot regions, where the intensity of the electromagnetic field is enhanced (see Supplementary Information Section S5 for a simulation of electromagnetic field localizations generated in a random system); this occurrence could lead to stronger emission properties in the system. Our Si NW samples exhibit efficient room temperature PL due to quantum confinement effects, as reported recently^26, 46^. In particular, the PL emission in sample 1 is strongly evident, even by the naked eyes^26^. Figure 4a shows the optical emission spectrum obtained by exciting the investigated sample with the 488 nm line of a laser. The PL band, peaking at 690 nm, is clearly visible. However, a further dominant feature is represented by an exceptionally strong first-order Si Raman peak at 500.7 nm (~520 cm^−1^ of Raman shift). To reveal the nature of this intense Raman signal, we excited the samples 1 and 2 at different laser wavelengths and compared the spectra with those of a single-crystalline Si (*c*-Si) sample identical to the one used to fabricate the NWs.

Note that the Raman enhancement (RE) is calculated as the ratio between the integrated intensities of the Si NWs’ first-order Raman peak and those of bulk silicon in the same experimental conditions for each excitation wavelength considered. Because the volume of the material involved in the scattering processes is lower in the case of the NW structure, we normalize the RE to the probed volume of the sample (REV), considering only 40% of silicon. This normalization is in agreement with the aforementioned composition assumed (40% air voids, 40% silicon and 20% silicon oxide). However, this normalization underestimates the exact Raman enhancement in the NW material because the Raman radiation is also trapped and then strongly absorbed when involved in multiple scattering processes. Thus, we can confirm that the detected Raman signal is only a part of the Raman scattered radiation.

**Figure 5:**
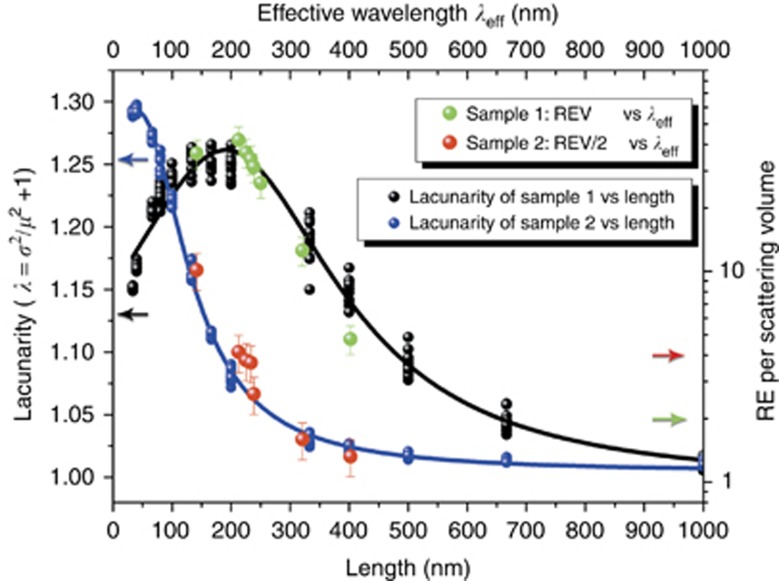
Comparison between lacunarity and Raman enhancement. The Raman enhancement of sample 1 (green dots) and sample 2 (red dots) plotted as a function of the effective incident laser wavelength propagating in the medium *λ*_eff_ and compared with the corresponding lacunarity curves (black and blue lines represent samples 1 and 2, respectively).

In Figure 4b, we show the trend of the REV of sample 1 as a function of the excitation laser wavelength *λ*, while its PL intensity at 690 nm, as excited by the different laser colors (PLE), is plotted in Figure 4c. The trend of the Raman enhancement as well as that of PLE reproduces with good agreement the ‘apparent absorbance’ shape of the Si NW fractal array. To extract the actual absorption from the NW layer, we calculate the apparent absorbance as obtained from the reflectance *R*_NW_% measurements as log(100/*R*_NW_%) after normalization to the reflectance of the silicon substrate. This curve represents the extinction signal due to in-plane multiple scattering processes through the 2D fractal structure, showing a maximum when the effective wavelength *λ*_eff_ resonantly matches the maximum intensity of the refractive index fluctuations in the medium (peak of lacunarity). When this occurrence is fulfilled, the in-plane multiple scattering processes in the fractal array can become more relevant. Exactly the same procedure was applied to elaborate the sample 2 data; the comparison between the corresponding apparent absorbance spectrum and REV is shown in Figure 4d. Thus, increasing the dwell time of the pump inside the material leads to the direct consequence of the increment of emission cross sections. A clear confirmation of this scenario is presented in Figure 5. Here, lacunarity curves for samples 1 and 2 are presented compared with the REV trends plotted as a function of *λ*_eff_; a quite perfect matching provides evidence regarding how the heterogeneities influence the scattering inside the sample structure. In particular, the results highlight how the Raman enhancement is correlated on the all length scales, which is characteristic of the finite fractal structure; note that lacunarity, which describes the refractive index fluctuations, is directly related to the scattering cross-section.

## Conclusions

In conclusion, we realized a forest of vertically aligned densely packed Si NWs arranged in a novel planar random fractal geometry by using an inexpensive, maskless and industrial compatible method. We demonstrated the strong correlation between the optical properties and the fractal characteristics. In fact, the fractal array promotes a high light-trapping efficiency with total reflectance values down to the 0.1% when the incident wavelength matches the maximum heterogeneity size exhibited by the arrangement of Si NWs. Furthermore, a strongly enhanced Raman emission, due to multiple scattering processes, is shown to depend on the effective wavelength resonantly matching the heterogeneity sizes of the Si NW 2D fractal arrangement. The observed performances make this novel 2D random fractal architecture among the most promising silicon-based materials for photovoltaic and photonic applications.

## References

[bib1] Wiersma DS. Disordered photonics. Nat Photon 2013; 7: 188–196.

[bib2] Brongersma ML, Cui Y, Fan SH. Light management for photovoltaics using high-index nanostructures. Nat Mater 2014; 13: 451–460. 24751773 10.1038/nmat3921

[bib3] Polman A, Atwater HA. Photonic design principles for ultrahigh-efficiency photovoltaics. Nat Mater 2012; 11: 174–177. 22349847 10.1038/nmat3263

[bib4] Atwater HA, Polman A. Plasmonics for improved photovoltaic devices. Nat Mater 2010; 9: 205–213. 20168344 10.1038/nmat2629

[bib5] Huang YF, Chattopadhyay S, Jen YJ, Peng CY, Liu TA, et al. Improved broadband and quasi-omnidirectional anti-reflection properties with biomimetic silicon nanostructures. Nat Nanotechnol 2007; 2: 770–774. 18654429 10.1038/nnano.2007.389

[bib6] Vynck K, Burresi M, Riboli F, Wiersma DS. Photon management in two-dimensional disordered media. Nat Mater 2012; 11: 1017–1022. 23042416 10.1038/nmat3442

[bib7] Riboli F, Caselli N, Vignolini S, Intonti F, Vynck K, et al. Engineering of light confinement in strongly scattering disordered media. Nat Mater 2014; 13: 720–725. 24836733 10.1038/nmat3966

[bib8] Yablonovitch E. Statistical ray optics. J Opt Soc Am 1982; 72: 899–907.

[bib9] Callahan DM, Munday JN, Atwater HA. Solar cell light trapping beyond the ray optic limit. Nano Lett 2012; 12: 214–218. 22149061 10.1021/nl203351k

[bib10] Yu ZF, Raman A, Fan SH. Fundamental limit of nanophotonic light trapping in solar cells. Proc Natl Acad Sci USA 2010; 12: 17491–17496. 10.1073/pnas.1008296107 PMC2955111 20876131

[bib11] Svensson T, Vynck K, Grisi M, Savo R, Burresi M et al. Holey random walks: optics of heterogeneous turbid composites. Phys Rev E 2013; 87: 022120. 10.1103/PhysRevE.87.022120 23496473

[bib12] Shalaev VM, Moskovits M, Golubentsev AA, John S. Scattering and localization of light on fractals. Physica A 1992; 191: 352–357.

[bib13] Podolskiy VA, Shalaev VM. Giant optical responses in microcavity-fractal composites. Laser Phys 2001; 11: 26–30.

[bib14] Krachmalnicoff V, Castanié E, De Wilde Y, Carminati R. Fluctuations of the local density of states probe localized surface plasmons on disordered metal films. Phys Rev Lett 2010; 105: 183901. 21231105 10.1103/PhysRevLett.105.183901

[bib15] Zhu LH, Shao MR, Peng RW, Fan RH, Huang XR et al. Broadband absorption and efficiency enhancement of an ultra-thin silicon solar cell with a plasmonic fractal. Opt Express 2013; 21: A313–A323. 24104419 10.1364/OE.21.00A313

[bib16] Albella P, Poyli MA, Schmidt MK, Maier SA, Moreno F et al. Low-loss electric and magnetic field-enhanced spectroscopy with subwavelength silicon dimers. J Phys Chem C 2013; 117: 13573–13584.

[bib17] Muskens OL, Rivas JG, Algra RE, Bakkers EPAM, Lagendijk A. Design of light scattering in nanowire materials for photovoltaic applications. Nano Lett 2008; 8: 2638–2642. 18700806 10.1021/nl0808076

[bib18] Diedenhofen SL, Janssen OTA, Grzela G, Bakkers EPAM, Rivas JG. Strong geometrical dependence of the absorption of light in arrays of semiconductor nanowires. ACS Nano 2011; 5: 2316–2323. 21366282 10.1021/nn103596n

[bib19] Strudley T, Zehender T, Blejean C, Bakkers EPAM, Muskens OL. Mesoscopic light transport by very strong collective multiple scattering in nanowire mats. Nat Photon 2013; 7: 413–418.

[bib20] Zou Y, Sheng X, Xia K, Fu HY, Hu JJ. Parasitic loss suppression in photonic and plasmonic photovoltaic light trapping structures. Opt Express 2014; 22: A1197–A1202. 24978082 10.1364/OE.22.0A1197

[bib21] Liu XG, Coxon PR, Peters M, Hoex B, Cole JM et al. Black silicon: fabrication methods, properties and solar energy applications. Energy Environ Sci 2014; 7: 3223–3263.

[bib22] Garnett E, Yang PD. Light trapping in silicon nanowire solar cells. Nano Lett 2010; 10: 1082–1087. 20108969 10.1021/nl100161z

[bib23] Garnett E, Brongersma ML, Cui Y, McGehee MD. Nanowire solar cells. Annu Rev Mater Res 2011; 41: 269–295.

[bib24] Burresi M, Pratesi F, Riboli F, Wiersma DS. Complex photonic structures for light harvesting. Adv Opt Mater 2015; 3: 722–743. 26640755 10.1002/adom.201400514 PMC4662022

[bib25] Tsakalakos L, Balch J, Fronheiser J, Korevaar BA, Sulima O et al. Silicon nanowire solar cells. Appl Phys Lett 2007; 91: 233117.

[bib26] Irrera A, Artoni P, Iacona F, Pecora EF, Franzò G, et al. Quantum confinement and electroluminescence in ultrathin silicon nanowires fabricated by a maskless etching technique. Nanotechnology 2012; 23: 075204. 22273546 10.1088/0957-4484/23/7/075204

[bib27] Bunde A, Havlin S. Fractals and Disordered Systems. 2nd ed. Berlin Heidelberg: Springer-Verlag. 1996.

[bib28] Rammal R, Toulouse G. Random walks on fractals structures and percolation clusters. J Phys Lett 1983; 44: L13–L22.

[bib29] Cazé A, Pierrat R, Carminati R. Spatial coherence in complex photonic and plasmonic systems. Phys Rev Lett 2013; 110: 063903. 23432244 10.1103/PhysRevLett.110.063903

[bib30] Karperien A. FracLac Is for Digital Image Analysis 1999–2013. Available at http://rsb.info.nih.gov/ij/plugins/fraclac/FLHelp/Introduction.htm.

[bib31] Voss RF, Laibowitz RB, Alessandrini EI. Percolation and fractal properties of thin gold films. In: Hughes BD, Ninham BW, editors. The Mathematics and Physics of Disordered Media: Percolation, Random Walk, Modeling and Simulation. Lecture Notes in Mathematics, vol. 1035. Berlin Heidelberg: Springer. 1983; 153–168.

[bib32] Plotnick RE, Gardner RH, Hargrove WW, Prestegaard K, Perlmutter M. Lacunarity analysis: a general technique for the analysis of spatial patterns. Phys Rev E 1996; 53: 5461–5468. 10.1103/physreve.53.5461 9964879

[bib33] Andersson TG. The initial growth of vapour deposited gold films. Gold Bull 1982; 15: 7–18.

[bib34] Holmberg VC, Bogart TD, Chockla AM, Hessel CM, Korgel BA. Optical properties of silicon and germanium nanowire fabric. J Phys Chem C 2012; 116: 22486–22491.

[bib35] Bruggemann DAG. Berechnung verschiedener physikalischer Konstanten von heterogen substanzen. Ann Phys 1935; 24: 636–679.

[bib36] Aspnes DE, Studna AA. Dielectric functions and optical parameters of Si, Ge, GaP, GaAs, GaSb, InP, InAs, and InSb from 1.5 to 6.0 eV. Phys Rev B 1983; 27: 985–1009.

[bib37] Waterman PC. Symmetry, unitarity, and geometry in electromagnetic scattering. Phys Rev D 1971; 3: 825–839.

[bib38] Borghese F, Denti P, Saija R, Toscano G, Sindoni OI. Multiple electromagnetic scattering from a cluster of spheres. I Theory. Aerosol Sci Technol 1984; 3: 227–235.

[bib39] Borghese F, Denti P, Saija R. Scattering from Model Nonspherical Particles. 2nd ed. Berlin Heidelberg: Springer-Verlag. 2007.

[bib40] Mishchenko MI, Travis LD, Lacis AA. Scattering, Absorption, and Emission of Light by Small Particles. Cambridge: Cambridge University Press. 2002.

[bib41] Kuga Y, Ishimaru A. Retroreflectance from a dense distribution of spherical particles. J Opt Soc Am A 1984; 8: 831–835.

[bib42] Van Albada MP, Lagendijk A. Observation of weak localization of light in a random medium. Phys Rev Lett 1985; 55: 2692–2695. 10032213 10.1103/PhysRevLett.55.2692

[bib43] Wolf PE, Maret G. Weak localization and coherent backscattering of photons in disordered media. Phys Rev Lett 1985; 55: 2696–2698. 10032214 10.1103/PhysRevLett.55.2696

[bib44] Muskens OL, Diedenhofen SL, Kaas BC, Algra RE, Bakkers EPAM et al. Large photonic strength of highly tunable resonant nanowire materials. Nano Lett 2009; 9: 930–934. 19193115 10.1021/nl802580r

[bib45] Stockman MI. Inhomogeneous eigenmode localization, chaos, and correlations in large disordered clusters. Phys Rev E 1997; 56: 6494–6507.

[bib46] Priolo F, Gregorkiewicz T, Galli M, Krauss TF. Silicon nanostructures for photonics and photovoltaics. Nat Nanotech 2014; 9: 19–32. 10.1038/nnano.2013.271 24390564

