# Supplementary material for: Strongly enhanced light trapping in a two-dimensional silicon nanowire random fractal array
Source: Light Sci Appl. 2016 Apr 8;5(4):e16062–. doi: 10.1038/lsa.2016.62 (PMC6059951; doi:10.1038/lsa.2016.62)
Supplement: Supplementary Information [file lsa201662x1.doc]

**Supplementary Information for**

**Strongly Enhanced Light Trapping in a Two-dimensional Silicon Nanowire Random Fractal Array**

Barbara Fazio ^1*^, Pietro Artoni^2^, Maria Antonia Iatì ^1^, Cristiano D’Andrea ^3^ , Maria Josè Lo Faro^1,2,3^, Salvatore Del Sorbo^4^, Stefano Pirotta^4^, Pietro Giuseppe Gucciardi^1^, Paolo Musumeci^2,3^, Cirino Salvatore Vasi ^1^, Rosalba Saija ^5^, Matteo Galli ^4^,Francesco Priolo^2,3,6^, and Alessia Irrera^1*^

*^1^* IPCF-CNR, viale F. Stagno d’Alcontres 37, Faro Superiore, 98158 Messina, Italy.

*^2^*Dipartimento di Fisica e Astronomia, Università di Catania, via S. Sofia, 64, 95123 Catania, Italy.

*^3^* MATIS IMM-CNR, via S. Sofia, 64, 95123 Catania, Italy.

*^4^* Dipartimento di Fisica, Università degli Studi di Pavia, via Bassi 6, 27100 Pavia, Italy.

*^5^*Dipartimentodi scienze matematiche e informatiche, scienze fisiche e scienze della terra, Università di Messina, I-98166 Messina, Italy

*^6^* Scuola Superiore di Catania,Università di Catania, via Valdisavoia, 9, 95123 Catania, Italy.

***Corresponding authors:** e-mail address: [fazio@ipcf.cnr.it](mailto:fazio@ipcf.cnr.it) , Tel.: +39 090 39762246; fax: +39 090 3974130;e-mail address: [irrera@ipcf.cnr.it](mailto:irrera@ipcf.cnr.it), Tel.: +39 090 39762266; fax: +39 090 3974130.

**Section S1. Model Building Cell method**

The planar arrangement of the NWs was also studied by using a bottom-up approach consisting in mapping the two-dimensional array images in figure 1b, c, and d (main text) through the iterative repetition of a planar Model Building Cell (MBC). The MBC, shown in figure S1.1, is set by matching the image in figure 1d (main text), which we assume as our elementary cell (80 nm x 70 nm) and shows a filling factor of 0.6 in agreement with the measured filling factor (see main text, figure 1e). The MBC is repeated until the entire area of interest has been covered, mapping the image in figure 1c (800 nm x 700 nm) and in figure 1b (8000 nm x 7000 nm) of the main text. The MBC is obtained considering the geometrical cross section of an aggregate of N=80 spheres, each with a radius r_0_=4 nm.


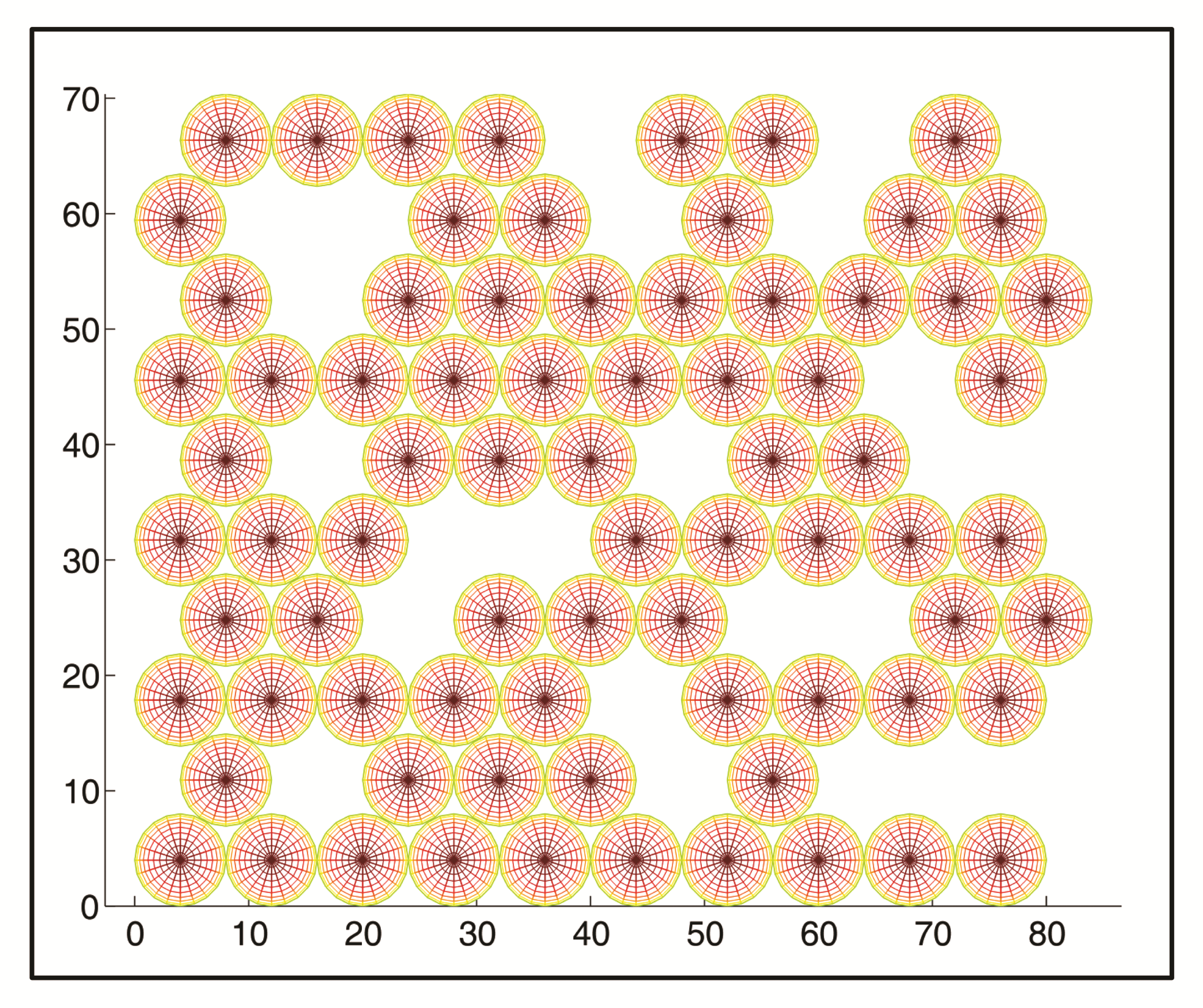


**Figure S1.1** Planar Model Building Cell used to calculate the fractal dimension of the sample.

This choice is suggested by micro-Raman results and transmission electron microscopy (TEM) images, which estimate the diameter of the NWs to be 7±2 nm. The most relevant parameter in the fractal characterization of an aggregate of N monomers is the fractal dimension[[1]](#endnote-2) *D*, defined by the relation[[2]](#endnote-3) $N\approx r_{g}^{D}$ where *r_g_* is the radius of gyration, expressed as

$r_{g}^{2}=\frac{1}{2N^{2}}\sum_{ij}^{N} (r_{i}-r_{j})^{2}$ (1)

with ***r***_i_ and ***r***_j_ position vectors of the *i*_th_ and *j*_th_ constituent monomers[[3]](#endnote-4). The radius of gyration for our MBC comes out to be *r_g0_* = 30.61 nm. We obtain the fractal dimension as the slope of the curve of log *N*_i_ vs log (*r_gi_*/*r_g0_)*, where *N*_i_ is the number of MBC which we need for an optimal coverage of the SEM image at the three different magnifications (figure 1b, c, and d of the main text) and *r_gi_* are the corresponding radii of gyration.

**Section S2. Lacunarity**

Lacunarity is an important parameter for the characterization of a fractal sample; it is the complementary information to the fractal dimension describing how the fractal texture is organized. It is closely related to the size distribution of the holes. Indeed, if a fractal has large holes, including large regions of space, it has high lacunarity. On the other hand, since lacunarity is related to the deviation of fractals from translational invariance[[4]](#endnote-5), it is expected to be high especially in random fractals.

The lacunarity of the Si NWs samples is calculated from the pixel distribution of the images shown in figure 2a and b of the main text and it is based on variation in pixels density at different box sizes during the standard fixed non-overlapping box counting scan. In particular, the number of pixels that are significant in describing the structure (the black ones in the inset of figure 2a) for each *ε* -sized box in a grid placed on the image is counted during the scan. These pixels have been chosen with a threshold level (a different threshold level does not affect the measure of the lacunarity). For each grid of dimension *ε*, the standard deviation *σ* and the mean number of pixels per box, *μ*, are calculated; the lacunarity values λ are defined as λ=(σ/μ)^2^. Thus, for each *ε* in each series of grid sizes, for each g, grid position, in a set of grid positions used to investigate the image, there will be a value of λ. As a consequence, there will never be a single "λ", but instead λ*_ε_*_, g_, and then more values for each grid (the black and blue dots in figure 2d). For each orientation of a series of grid sizes there will be a λ_g_ value that represents the lacunarity; it is extracted by the slope ln-ln regression line of λ(*ε*)*. *Note that, when an image is totally homogeneous, no variation in the pixel box will be detected; σ=0 and as a consequence λ=0. This means that the slope of the ln(λ)-ln(*ε*) regression line will be undefined. For this reason the data are transformed by using (λ+1), indeed, in this case the totally homogeneous image has a slope of 0, meaning the absence of gaps.

Since the lacunarity dependence with the box size can be very different depending on the calculation method[[5]](#endnote-6),[[6]](#endnote-7), for the sake of comparison, we calculated the lacunarity related to the sample 1 and sample 2 by means of another methodology. In this different sampling approach we calculate the number of pixels in a grid placed on the respective images during the overlapping sliding box counting. The lacunarity curves of the two samples obtained with the two methods differ slightly in the magnitude of the curve and very little in its shape. In figure S2.1 the results obtained by both the non-overlapping box counting analysis and the overlapping sliding box analysis are compared.


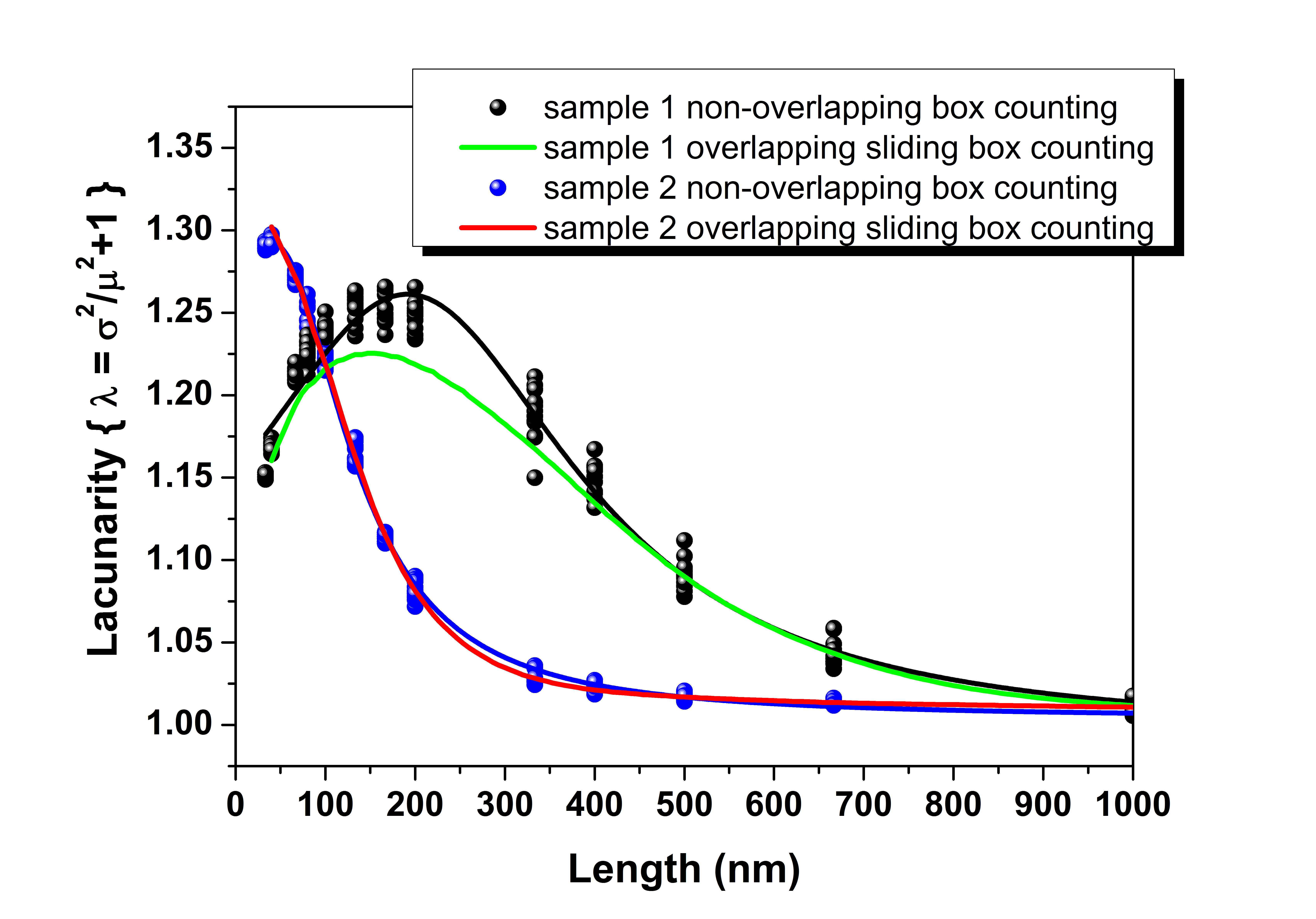


**Figure S2.1 Comparison between lacunarity curves obtained with different approaches.** Lacunarity curves of sample 1 (black and green) and sample 2 (blue and red) obtained both with “non-overlapping”(black and blue) and “overlapping sliding”(green and red) box counting methods respectively.

**Section S3. Total reflectance pattern: a numerical investigation**

We realize a model structure to theoretically investigate the effect of the sample heterogeneity on the total reflectance pattern (see figure S3.1). We choose to model the system as a layer of randomly distributed spherical cavities (n=1) embedded in a homogeneous matrix with an effective refractive index ranging between n=2.57 and n=1.97 (spanning from an incident wavelength of 360 nm to 633 nm), as obtained from the Bruggeman mixing rule (see the main text for details). In our numerical simulations we investigate both, the effect of the cavity size and of the edge-to-edge distance between close cavities. We run computations assuming the presence of a different number of cavities and we finally chose to adopt a cavity number equal to 18, as a good compromise between the need to simulate the sample complexity and the convenience to deal with a reasonable computational time.


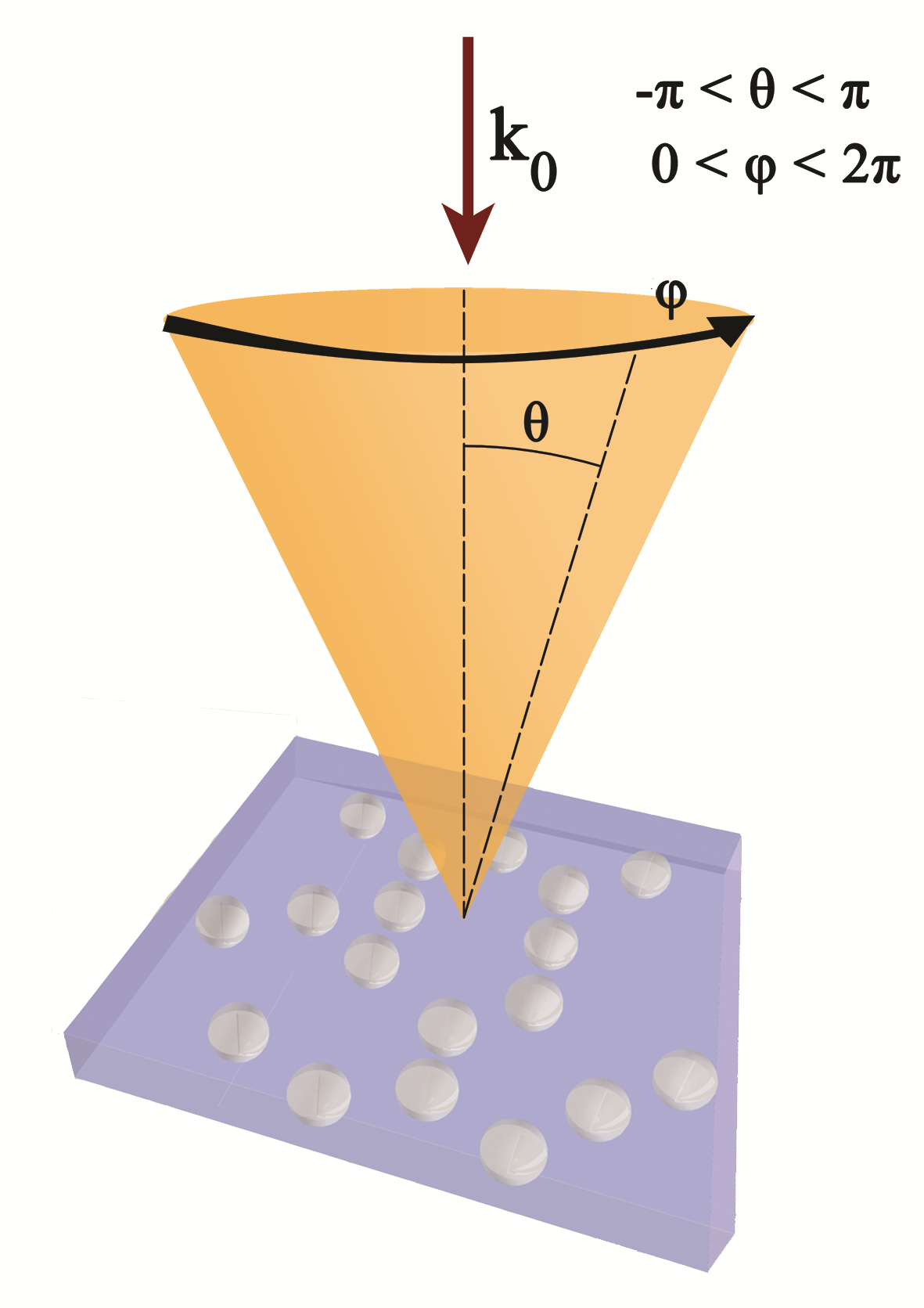


**Figure S3.1. Sketch of the model structure used to compute the total reflectance pattern.** The model structure is formed by 18 randomly distributed spherical air cavities embedded in a homogeneous layer. In the total reflectance computations we sum up the intensities of the fields scattered out of the plane spanning the half-space containing the incident wave vector.

Our computations have been performed using the Transition matrix approach, based on the electromagnetic multipole field expansion[[7]](#endnote-8),[[8]](#endnote-9),[[9]](#endnote-10). The elements of the Transition matrix encompass all information about particle size, morphology and composition, but are independent of the propagation direction and polarization state of the incident and of the scattered fields. Therefore, the Transition matrix has to be computed only once and then can be used in calculations for any direction of incidence and scattering and for any polarization state of the incident field[[10]](#endnote-11).

First we explore the effect of the size cavities, assuming cavity diameters ranging from 160 to 200 nm and an edge-to-edge distance *l* between nearest cavities in a range between where λ is the value corresponding to the minimum of the experimental reflectance curve (428 nm). The choice of these values has been motivated by the convenience to explore a size range comparable with the length scales over which the lacunarity has its maximum value in sample 1 (see main text, figure 2d). In each simulation we assume that all the cavities have the same diameters. The incident field propagates normally to the plane where the model structure is placed. In figure S3.2 we show the total reflectance pattern corresponding to a cavity diameter of 160 nm (blue line) and of 200 nm (red line) compared with the computed silicon bulk (without holes) reflectance (solid black line) and the experimental reflectance pattern (dotted line); in this computations the effective refractive index is fixed at 2.57. The total reflectance is computed summing up the intensities of the fields scattered out of the model structure plane at any angle, i.e. spanning the half-space containing the incident wave vector (as shown in figure S3.1). We see from figure S3.2 that, as the cavity diameter increases and as a consequence the edge-to-edge distance between the nearest cavities decreases, the position of the minimum in the reflectance pattern blue shifts.

**Figure S3.2. Total reflectance pattern of the model structure for different cavity diameters.** Total reflectance simulated for a model structure having cavity diameter *d* fixed at 160 nm (blue line) and at 200 nm (red line); the effective refractive index is fixed at 2.57. The calculated curves are compared to the experimental curve (dotted line). The black line represents the calculated reflectance for silicon bulk without holes.

Note that in the computations we assume that all the cavities have the same diameter, while the experimental sample is characterized by a fractal size distribution ranging over all the length scales. In figure S3.3 we further explore the effect of the edge-to-edge distance keeping the cavity diameter fixed at 190 nm. We show the reflectance pattern corresponding to an edge-to-edge distance equal to the incident wavelength λ corresponding to the minimum of the experimental reflectance curve (428 nm, green line), and close to the respective effective wavelength λ_eff_ (about 190 nm, blue line) as defined in the paper main text. Again, the refractive index is fixed at n=2.57. The results confirm as the reflectance pattern is sensitive to changes in the edge-to-edge distance.

**Figure S3.3. Total reflectance pattern of the model structure for different edge-to-edge cavity distances.** Total reflectance simulated for a model structure having cavity diameter *d* fixed at 190 nm and edge-to-edge distances equal to the incident wavelength λ (428 nm, green line) and close to the corresponding effective wavelength in the medium λ_eff_ (190 nm, red line). The effective refractive index is fixed at n=2.57. The calculated curves are compared to the experimental (dotted line). The black solid line represents the calculated reflectance for silicon bulk without holes.

In figure S3.4 we consider the material refractive index dependence on the wavelength and how it affects the reflectance. We fix both the cavity diameter and the edge-to-edge distance between the nearest cavities at 190 nm. Now we compare the computed reflectance curve at fixed refractive index (n=2.57; red curve) with that one obtained with an effective refractive index changing across the range n=2.57 and n=1.97 (spanning from an incident wavelength of 360 nm to 633 nm; blue curve). The figure shows that introducing the wavelength refractive index dependence, we get a more realistic description of the sample and thus a better matching with the experimental curve.

**Figure S3.4. Total reflectance pattern of the model structure assuming a wavelength refractive index dependence.** Total reflectance simulated for a model structure having both cavity diameter *d* and edge-to-edge distance fixed at 190 nm. Red line: refractive index fixed at n=2.57; blue line: refractive index ranging between n=2.57 and n=1.97. The calculated curves are compared to the experimental (dotted line).

**Section S4. Specular reflectance**

The specular reflectance of sample 1 as a function of the incidence angle is evaluated in a spectral range between 300 and 800 nm and plotted in figure S4.1 at the wavelengths of 428 nm, 532 nm and 680 nm (as representative of the minimum of the reflectance spectrum, the green and the red spectral regions, respectively). The angle–resolved measurements have been acquired with a home-made micro-reflectometer coupled to a Fourier-transform spectrometer (Bruker IFS 66s), for incidence angles varying in the range 0-60 degrees. Absolute reflectance values were independently calibrated using a stabilized He-Ne laser at λ=632.8 nm wavelength.

Through all the investigated spectral range the specular reflectance shows a minimum when the incident radiation is along the wire long axis and then slowly increases after 10° remaining largely lower than the 1% until 60°. This result is crucial for the use of this material in photovoltaic application, because it ensures high light trapping efficiency along all the day, since it is not strongly affected by the direction of the solar rays on the device.


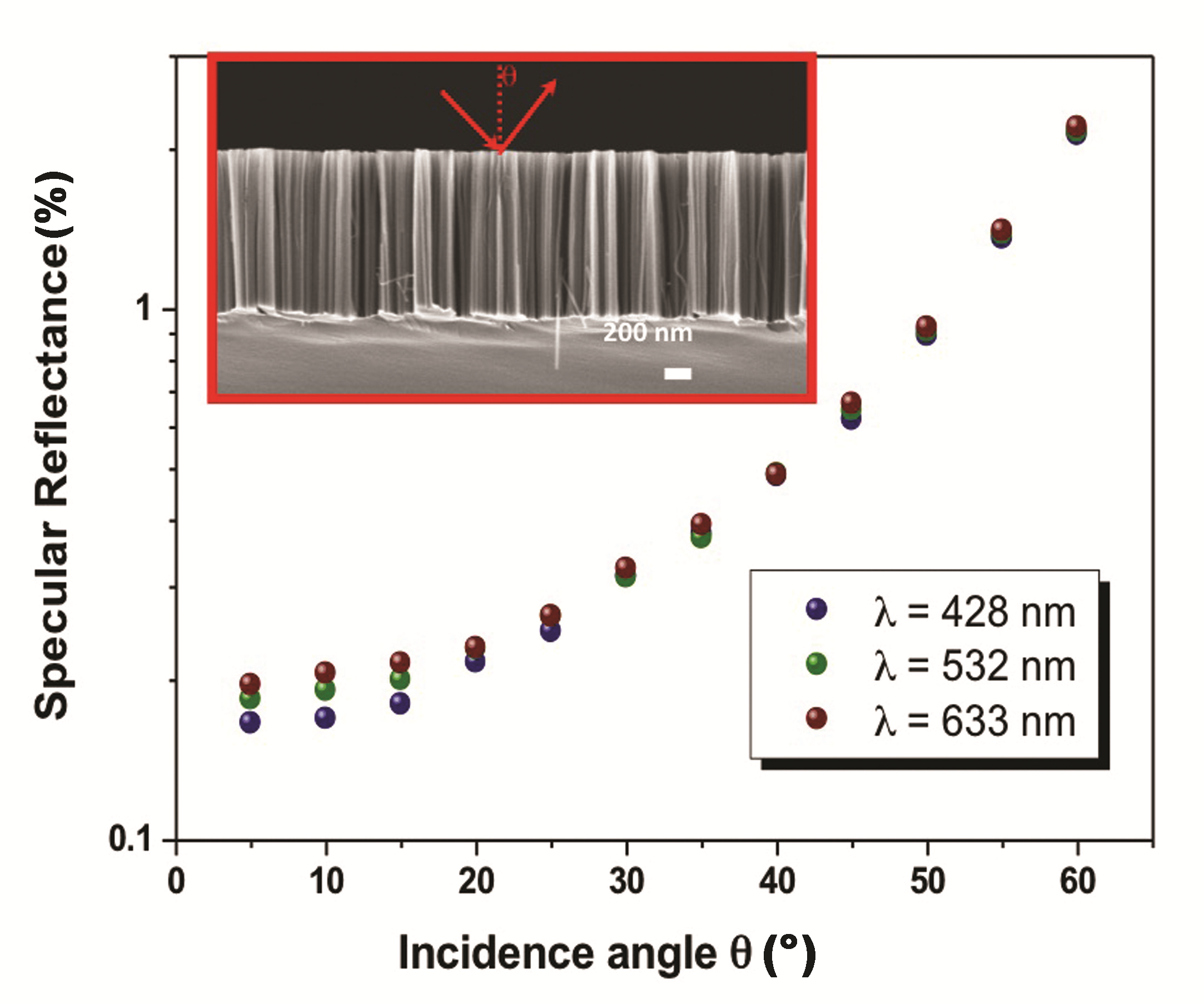


**Figure S4.1. Specular reflectance as a function of the incidence angle**. Measurements with non-polarized light at a wavelength of 428 nm (blue dots), 532 nm (green dots) and 633 nm (red dots). In the inset a sample cross section with an experimental scheme that illustrates the incident and scattered light (red arrows) forming a θ angle with the normal to the sample surface.

**Section S5. Hot spot formation in random systems**

We perform a simulation to calculate the intensity distribution map of the scattered light in the near field zone by a random array of nanospheres. We simulate the system as a collection of 300 nanospherical particles, each with a radius of 40 nm (panel a); the choice for the particle dimensions and arrangement has been suggested by the opportunity to reproduce the heterogeneity size for which lacunarity is maximum in our nanowires material (sample 1 of the main text). We calculate the particle refractive index by a Bruggeman mixing rule assuming a composition of 70% Si and 30% SiO_2_. We plot in figure S5.1 the near field normalized intensity map distribution $\left|E_{sca}/E_{inc}\right|^{2}$with $E_{sca}$and $E_{inc}$amplitudes of the scattered and of the incident electric field, respectively. We calculate the near field maps for three different wavelengths of the incident radiation in the visible. In all the investigated cases we observe the formation of “hot spot” regions (much smaller than λ) where the local field is enhanced compared to the incident one. The distribution of these regions appears more localized and the field intensity is higher at shorter wavelength (panel b). As the wavelength increases (panel c and d) the hot spot regions appear more enlarged and delocalized along the structure.

Now, field localization occurring in random systems appears even more strikingly in those possessing dilational symmetries, namely fractals[[11]](#endnote-12). Thus, we can extend the results of this investigation to fractal models, for which the heterogeneity sizes are correlated on all length scales, between the size of constituent particles to that corresponding to the dimensions of the fractal. Hence, whatever the impinging wavelength, it is always possible to match it with the inhomogeneities length, and therefore with the refractive index fluctuations, generating a strong scattering that leads to interesting localization effects.


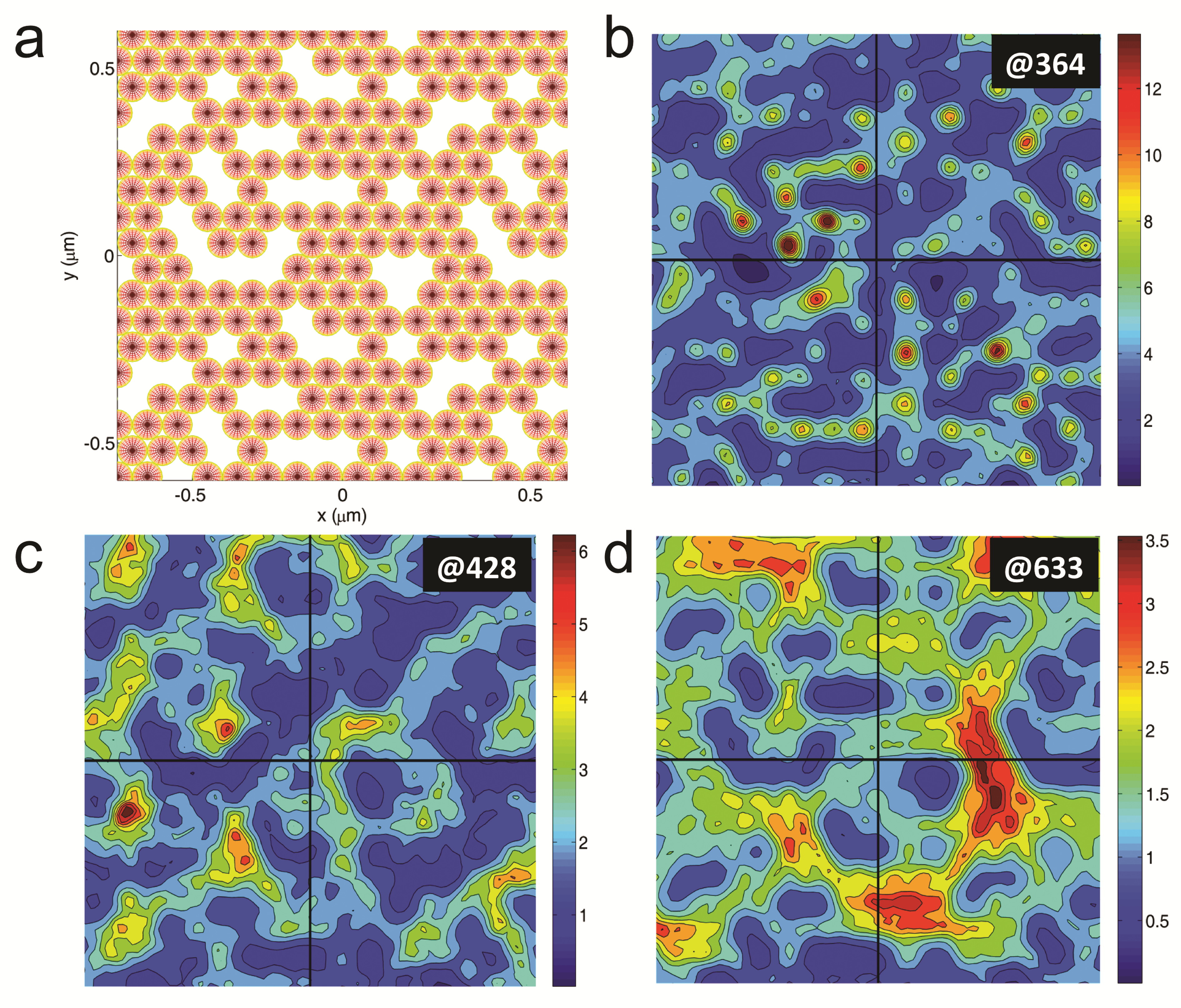


**Figure S5.1 Near field intensity map.** Model random structure (**a**). Maps of the normalized scattered field intensity distribution plotted as $\left|E_{sca}/E_{inc}\right|^{2}$, for an exciting field with wavelength 364 nm (**b**), 428 nm (**c**) and 633 nm (**d**).

**REFERENCES**

1. Witten TA, Cates ME. Tenuous structures from disorderly growth processes. *Science* 1986; **232**: 1607-1612. [↑](#endnote-ref-2)
2. Meakin P. Fractal scaling in thin film condensation and material surfaces. *CRC Critical Reviews in Solid State and Materials Science* 1987; **13**:143-189. [↑](#endnote-ref-3)
3. Mukai T, Ishimoto H, Kozasa T, Blum J, Greenberg JM. Radiation pressure forces of fluffy porous grains. *Astron Astrophys* 1992; **262**: 315-320. [↑](#endnote-ref-4)
4. Karperien A. FracLac for ImageJ . http://rsb.info.nih.gov/ij/plugins/fraclac/FLHelp/Introduction.htm. 1999-2013. [↑](#endnote-ref-5)
5. Dale MRT. Lacunarity analysis of spatial pattern: A comparison. *Landscape Ecology* 2000; **15**: 467-478. [↑](#endnote-ref-6)
6. Pendleton DE, Dathe A, Baveye P. Influence of image resolution and evaluation algorithm on estimates of the lacunarity of porous media. *Phys Rev E* 2005; **72**: 041306. [↑](#endnote-ref-7)
7. Waterman PC. Symmetry, unitarity, and geometry in electromagnetic scattering. *Phys Rev D* 1971; **3**: 825-839. [↑](#endnote-ref-8)
8. Borghese F, Denti P, Saija R, Toscano G, Sindoni OI. Multiple electromagnetic scattering from a cluster of spheres. I. Theory. *Aerosol Sci Technol* 1984; **3**: 227-235. [↑](#endnote-ref-9)
9. Borghese F, Denti P, Saija R. *Scattering from Model Nonspherical Particles.* 2nd edition. Berlin Heidelberg: Springer-Verlag; 2007. [↑](#endnote-ref-10)
10. Mishchenko MI, Travis LD, Lacis AA. *Scattering, Absorption, and Emission of Light by Small Particles*. Cambridge: Cambridge University Press; 2002. [↑](#endnote-ref-11)
11. Podolskiy VA, Shalaev VM. Giant optical responses in microcavity-fractal composites. *Laser Physics* 2001; **11**: 26-30. [↑](#endnote-ref-12)
